# Effect of the Monothiol Glutaredoxin GrxD on 2,4-Diacetylphloroglucinol Biosynthesis and Biocontrol Activity of *Pseudomonas fluorescens* 2P24

**DOI:** 10.3389/fmicb.2022.920793

**Published:** 2022-07-08

**Authors:** Qiuling Dong, Qing Yan, Bo Zhang, Li-qun Zhang, Xiaogang Wu

**Affiliations:** ^1^Guangxi Key Laboratory of Agro-Environment and Agro-Product Safety, College of Agriculture, Guangxi University, Nanning, China; ^2^Department of Plant Sciences and Plant Pathology, Montana State University, Bozeman, MT, United States; ^3^College of Plant Protection, China Agricultural University, Beijing, China

**Keywords:** *Pseudomonas fluorescens*, GrxD, RsmA/RsmE, PhlF, 2,4-DAPG

## Abstract

*Pseudomonas fluorescens* 2P24 is a plant root-associated bacterium that suppresses several soilborne plant diseases due to its production of the antibiotic 2,4-diacetylphloroglucinol (2,4-DAPG). The biosynthesis of 2,4-DAPG is controlled by many regulatory elements, including the global regulator of the Gac/Rsm regulon and the pathway-specific repressor PhlF. In this work, a novel genetic element *grxD*, which encodes the monothiol glutaredoxin GrxD, was identified and characterized in the production of 2,4-DAPG in *P. fluorescens* 2P24. Our data showed that the mutation of *grxD* remarkably decreased 2,4-DAPG production. GrxD lost its ability to alter the production of 2,4-DAPG when the active-site CGFS motif of GrxD was mutated by site-directed mutagenesis. Further studies showed that the RsmA and RsmE proteins were essential for the GrxD-mediated regulation of 2,4-DAPG and exoprotease production. In addition, our data revealed that the deletion of *grxD* increased the expression of *phlF*, which negatively regulated the production of 2,4-DAPG. In addition, the *grxD* mutant was severely impaired in the biocontrol effect against the bacterial wilt of tomato. Overall, our results indicated that the monothiol glutaredoxin GrxD is involved in the production of 2,4-DAPG of *P. fluorescens* by influencing the Gac/Rsm global signaling pathway and transcriptional regulator PhlF and is essential for the biocontrol properties.

## Introduction

Certain strains of *Pseudomonas*, known as plant growth-promoting rhizobacteria (PGPR), can competitively colonize plant roots, enhance crop growth, and produce antibiotics to control plant diseases (Haas and Défago, 2005). Efficient root colonization by these rhizobacteria is an essential prerequisite for the biocontrol of root pathogens (Benizri et al., 2001). However, microbial colonization by PGPR is a highly complex process and is involved in the interactions among plants, rhizobacteria, and environmental factors. During the early phases of root colonization, some PGPR penetrates the intercellular spaces of host plants, which trigger microbe-associated molecular patterns similar to those of pathogenetic fungi (Pršiæ and Ongena, 2020). This recognition of host cells then activates an oxidative burst (or respiratory burst), leading to the release of reactive oxygen species (ROS) (Segonzac and Zipfel, 2011). Besides the plant-derived ROS, rhizobacteria must cope with the self-made ROS produced as a byproduct of aerobic respiration or environmental cues (Nanda et al., 2010). To counteract the damages caused by ROS, rhizobacteria possess multiple antioxidant mechanisms, such as glutathione (GSH)- and thioredoxin (TRX)-dependent systems, catalases, and superoxide dismutases (Forman et al., 2009; Heck et al., 2010).

The GSH-dependent system, which is also called the glutaredoxin (GRX) system, consists of GRX, GSH, NADPH, and GSH reductase, and controls the cellular redox state of a myriad of proteins together with thioredoxins (Fernandes and Holmgren, 2004). GRXs represent a group of ubiquitous oxidoreductases of the thioredoxin superfamily and are widely conserved in most eukaryotes and prokaryotes. These proteins preferentially reduce the disulfide bonds formed between the cysteine residues of glutathione and its target proteins. GRX family proteins are required for many biological processes, including iron–sulfur cluster (Fe–S) biogenesis, iron homeostasis, symbiotic capacity, cell survival, and production of virulence factors (Meyer et al., 2009; Poor et al., 2014).

Many GRX isoforms have been identified and characterized by different bacteria. On the basis of their structures and catalytic properties, GRXs are divided into two categories: dithiol (C [PG/FY] C motif) and monothiol (CGFS motif) GRXs. The dithiol GRX is discovered in *Escherichia coli* as an alternative donor to TRXs for ribonucleotide reductase (Holmgren, 1976). Dithiol GRXs Grx1 and Grx3 are small proteins (∼10 kDa) with the CPYC motif as their active site. Grx1 is reported to influence the intracellular disulfides of ribonucleotide reductase Ia and 3′-phophoasenylyl sulfate, whereas Grx3 has only 5% of the catalytic activity of Grx1 for ribonucleotide reductase Ia (Lilling et al., 1999). By contrast, monothiol GRXs have the CGFS motif as their active site and are proposed to function in the Fe–S cluster storage and delivery (Couturier et al., 2015).

Although GRXs have been investigated extensively for many years, knowledge about their functions in the biocontrol ability of PGPR remains limited. *Pseudomonas fluorescens* 2P24 is a root-associated beneficial bacterium from the rhizosphere of wheat. Strain 2P24 can efficiently colonize wheat roots and exhibits biocontrol activity against phytopathogens, such as *Rhizoctonia solani*, *Ralstonia solanacearum*, and *Gaeumannomyces graminis* var. *tritici* (Wei et al., 2004a). This biocontrol capacity relies on the production of secondary metabolite 2,4-diacetylphloroglucinol (2,4-DAPG) (Wei et al., 2004b). The biosynthetic cluster for 2,4-DAPG has been identified and characterized in strain 2P24 and other biocontrol pseudomonads (Bangera and Thomashow, 1999; Wu et al., 2010). The 2,4-DAPG biosynthetic gene cluster comprises *phlACBD* genes. *phlA* and *phlF* (encoding a pathway-specific regulator) genes are divergently transcribed (Abbs et al., 2002). The production of 2,4-DAPG by *P. fluorescens* is a dynamic process regulated by many regulatory elements. The Gac/Rsm signaling cascade is necessary for the production of 2,4-DAPG (Zhang et al., 2020a). This signaling cascade is initiated by the GacS/GacA two-component system. Upon stimulation, the GacS sensor kinase autophosphorylates and then activates the response regulator GacA by phosphotransfer, which directly regulates the expression of the non-coding small RNA (sRNA) genes, i.e., *rsmX1*, *rsmX*, *rsmY*, and *rsmZ*, in strain 2P24 (Heeb and Haas, 2001; Zhang et al., 2020a). These sRNAs can sequester CsrA family proteins RsmA and RsmE, which can repress the translation of *phlA* mRNA (Zhang et al., 2020b). Interestingly, GacA negatively controls the transcription of the sRNA gene *rgsA*, which negatively influences the production of 2,4-DAPG (Zhang et al., 2020a).

In this study, we screen the mutant libraries of *P. fluorescens* to identify genetic factors that influence the production of 2,4-DAPG. Our data indicate that the mutation of the cytosolic monothiol glutaredoxin-encoding gene *grxD* remarkably decreases the production of 2,4-DAPG. Further investigations reveal that the protein levels of RsmA/RsmE and PhlF are significantly increased in the *grxD* mutant. The mutation of *phlF*, *rsmA*, or *rsmE* genes in the *grxD* mutant background can improve the production of 2,4-DAPG. Furthermore, GrxD regulated the production of 2,4-DAPG and exoprotease by influencing the protein levels of RsmA and RsmE. Overall, this work reveals the essential role of *grxD* in redox homeostasis, siderophores, and biocontrol ability in *P. fluorescens*.

## Materials and Methods

### Strains and Culture Conditions

Bacterial strains and plasmids used in this work are listed in Supplementary Table 1. *Pseudomonas fluorescens* and *E. coli* strains were routinely grown in the Lysogeny Broth (LB) medium, King’s B medium (KB) (King et al., 1954), or ABM medium (Chilton et al., 1974) for general genetic procedures. *Rhizoctonia solani* A01 was routinely cultured in potato dextrose agar (PDA) medium. When required, antibiotics were used at final concentrations as follows: ampicillin (50 μg/ml), kanamycin (50 μg/ml), gentamicin (5 μg/ml), and tetracycline (20 μg/ml).

### Tn5 Transposon Mutagenesis

The Tn5-transposon mutagenesis was performed by conjugating a mini-Tn5 suicide plasmid pUT-Km into *P. fluorescens* 2P24, as previously described (Wu et al., 2010). Approximately 5,000 mutants were screened for attenuating the antifungal activity against *R. solani* A01. Mutants were grown in 96-well microtiter plates containing KB broth. Overnight cultures (2 μl) were then transferred to the OmniTray plate containing PDA with a suspension of blended fungal hyphae of *R. solani* A01. Mutants in which the antifungal activity was significantly decreased compared with the parental strain were selected, and the Tn5 insertion site was determined as previously described (Wu et al., 2010).

### Construction of Deletion Mutants and Complementary Strains

The *grxC*, *grxD*, *grxF*, and *grxG* genes were deleted by allelic replacement according to the method of Zhang et al. (2018). In-frame deletion plasmids in *grxC*, *grxD*, *grxF*, and *grxG* were created by overlapping PCR of the flanking regions of the target genes (primers are listed in Supplementary Table 2). The resulting DNA fragments were then cloned into the suicide vector p2P24Km (Zhang et al., 2018). The constructed plasmids p2P24Km-grxC, p2P24Km-grxD, p2P24Km-grxF, and p2P24Km-grxG were introduced into *P. fluorescens* 2P24 or its derivatives by the electroporation, and colonies with kanamycin sensitivity and sucrose resistance were selected and confirmed by PCR and verified by Sanger sequencing.

To generate complemented strains, the promoter and open reading frame regions of target genes were cloned into the broad-host range expression plasmid pRK415 (Keen et al., 1988). The resulting plasmids were confirmed by sequencing and introduced into *P. fluorescens* cells by electroporation. The successful construction was confirmed by sequencing and electroporation into *P. fluorescens* cells.

### Site-Directed Mutagenesis

Site-directed mutagenesis was accomplished using the site-directed mutagenesis Kit (Sangon Biotech, Shanghai, China) with the plasmid p415-grxD to substitute critical residue in the CGFS motif (from CGFS to SGFS) of GrxD. The substitution was confirmed by DNA sequencing.

### Antifungal Activity of *Pseudomonas fluorescens*

Antifungal activity of 2P24 and its derivatives were determined on the PDA agar plate against *R. solani* A01. A 0.6-cm *R. solani* A01 plug was inoculated in the center of the PDA agar plate. In total, 5 μl of bacterial cultures grown overnight were then spotted on the plate at a distance of 2.5 cm from the fungal colony. The plates were then incubated at 30°C for 36 h, and the growth inhibition of *R. solani* was assessed.

### Extraction and Detection of 2,4-DAPG and Phloroglucinol

The production of 2,4-DAPG by *P. fluorescens* 2P24 and its derivatives was measured after being cultured in 30 ml KB medium at 30°C for 24 h. 2,4-DAPG was analyzed in ethyl acetate extract of the culture supernatants using high-performance liquid chromatography (HPLC) as described by Shanahan et al. (1992). A direct colorimetric assay was performed for quantification of the concentration of PG using 0.2% 4-hydroxy-3-methoxycinnamaldehyde solution in ethanol/HCL (v/v 3:1). The optical density of the reaction products was then measured at 550 nm (Kidarsa et al., 2011).

### Phenotypic Assays

The swimming and swarming motilities of *P. fluorescens* 2P24 were tested on an LB plate as described previously (Ha et al., 2014). Overnight bacterial cultures were inoculated and adjusted to the same bacterial density. In total, 5 μl aliquots were then inoculated onto the center of the swimming and swarming plates, respectively. The diameter of swim zones was measured after 24 h at 30°C.

The production of siderophore was determined by chrome azurol S (CAS) assay (Schwyn and Neilands, 1987). In total, 5 μl of overnight bacterial cultures were spotted on the center of the CAS plate. The production of siderophore was determined by measuring the diameter of an orange zone after incubation at 30°C for 24 h.

To visualize the production of exoprotease production of strain 2P24 and its derivatives, 5 μL drops of overnight bacterial cultures were inoculated onto a 2.5% skimmed milk agar plate. A visible halo appeared on the plate was measured after 24 h of incubation at 30°C.

For quantification of iron concentration, overnight bacterial cultures were collected and resuspended with 1 ml H_2_O and 0.5 ml fresh iron-releasing buffer containing 0.142 M KMnO_4_ and 0.6 M HCl. The mixtures were incubated at 60°C for 2 h and then added with 100 μl iron-reaction buffer containing 13.1 mM neocuproine, 6.5 mM ferrozine, 2 M ascorbic acid, and 5 M ammonium acetate at 25°C for 0.5 h. The subsequent cultures were centrifuged, and the absorbance was determined at 562 nm. The iron concentration was calculated using a FeCl_3_ standard curve.

To assess survival in response to oxidative stress, *P. fluorescens* 2P24 and its derivatives were grown overnight in LB broth, and cells were collected and re-suspended in distilled H_2_O to obtain an OD at 600 nm of 1.0. Suspended cells were treated with 0.6 mM H_2_O_2_ and 5 mM CuOOH and incubated at 30°C for 1 h. Cell cultivability was assessed by plating serial dilutions of the cultures on ABM plates.

The *P. fluorescens* 2P24 and its derivatives were grown overnight in the LB broth and were adjusted to an optical density at 600 nm of 1.0. In total, 20 μl of the bacterial cultures were then inoculated into 20 ml ABM medium in Erlenmeyer flasks at 30°C with shaking at 180 rpm. The growth of bacteria was indicated by optical density at 600 nm.

### Western Blot Analyses

The *P. fluorescens* 2P24 and its derivatives were grown in LB at 30°C for 18 or 36 h and a 1 ml sample was taken. Cells were then suspended in phosphate-buffered saline (PBS) buffer and lysed by sonication. Samples containing 30 μg of total protein were separated by a 12% (vol/vol) sodium dodecyl sulfate polyacrylamide gel electrophoresis (SDS-PAGE) followed by Western blot analysis using an anti-Flag antibody (1:2,000) (Sangon) and anti-RNA polymerase monoclonal antibody (1:2,000) (Neoclone), respectively. The resulting blots were developed using the chemiluminescence detection kit (Thermo Fisher, Waltham, MA, United States).

### β-Galactosidase Activity Assay

*Pseudomonas fluorescens* cells were grown in BL broth at 30°C with shaking at 200 rpm. Cells were collected at different points of the growth curve. β-Galactosidase activities were then determined using the Miller method (Miller, 1972).

### Plant Disease Suppression Assay

For the plant assays, the surface sterilized tomato seeds were cultured on the Murashige and Skoog (MS) medium with vitamins in 1-L sterile vials. After germination, tomato seedlings were maintained under sterile conditions up to the four-leaf stage. Plants were then uprooted carefully, and the root tips of the plants were cut off. Injured roots were immersed in the culture suspension of *R. solanacearum* GX02 (concentration 1 × 10^7^ CFU per milliliter). One hour after infection, tomato seedlings were planted in separate flasks containing sterile soil. Soil inoculation was performed by mixing with sterile water or *P. fluorescens* strains at concentrations of 1 × 10^7^ CFU per gram of soil, respectively. At 7 or 14 days after inoculation, plant survival was determined. The disease index scale from 0 to 4 was determined as described previously (Liang et al., 2020): 0 – no wilt symptoms; 1 – wilt symptoms on 1–25% of the leaves; 2 – wilt symptoms on 26–50% of the leaves; 3 –wilt symptoms on 51–75% of the leaves; and 4 – wilt symptoms on more than 76% of the leaves.

### Statistical Analysis

The signification of differences was assessed with Student’s *t*-test. Asterisks or different letters indicated *p-*values (*p* < 0.05), and results were presented as the mean ± standard deviation. Each experiment was performed three times with similar results.

## Results

### Identification of the *grxD* Gene in *Pseudomonas fluorescens* 2P24

A Tn5 transposon mutant library was constructed, and ca. 5,000 mutants were screened for loss of inhibition against the fungal pathogen *R. solani* to identify novel regulators of the production of 2,4-DAPG in *P. fluorescens* 2P24. Six mutants were found to have a reduced ability to repress the mycelial growth of *R. solani* (Supplementary Table 3). One of the mutants, 2P24M-39, fully abolished its ability to inhibit the mycelial growth of *R. solani*. DNA sequence analysis showed that the Tn5 transposon was inserted into the C0J56_22945 gene, encoding the monothiol glutaredoxin GrxD (accession AUM71418.1).

An *in silico* analysis of the 2P24 genome identified three paralogs of GrxD, namely, GrxC (locus C0J56_02105, accession AUM67817.1), GrxF (locus C0J56_23915, accession AUM71586.1), and GrxG (locus C0J56_21615, accession AUM72833.1). Phylogenetic analysis revealed that these four proteins belonged to two GRX classes (data not shown). GrxD (113 aa, 12.12 kDa) is a class I GRX with the CGFS motif that is typical of monothiol GRXs, whereas GrxC (84 aa, 9.34 kDa), GrxF (118 aa, 13.4 kDa), and GrxG (123 aa, 14.16 kDa) belong to class II GRX, which contain the dithiol C(PG/FY)C active site.

### Effect of GRXs on the Production of 2,4-Diacetylphloroglucinol in *Pseudomonas fluorescens*

**FIGURE 1:**
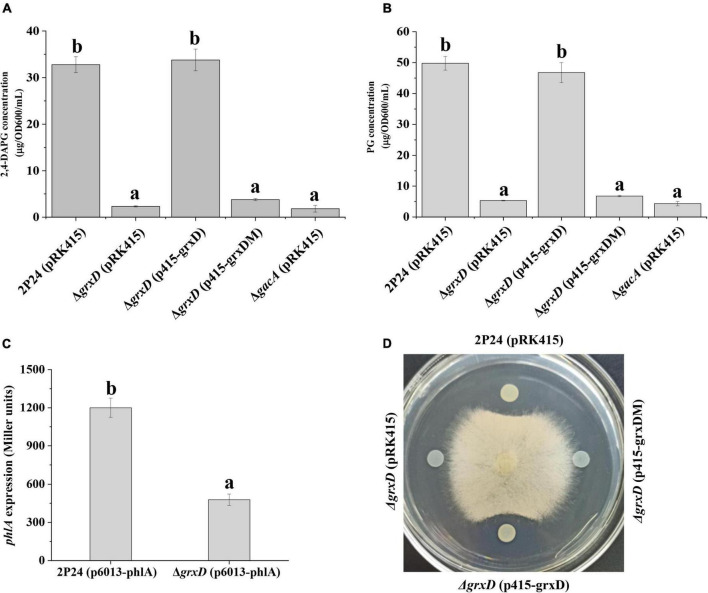
The effect of *grxD* gene on 2,4-diacetylphloroglucinol (2,4-DAPG) biosynthesis **(A)**, phloroglucinol (PG) production **(B)**, *phlA* expression **(C)**, and antifungal activity **(D)** of *Pseudomonas fluorescens* 2P24. **(A)** High-performance liquid chromatography (HPLC) analysis of the concentration of 2,4-DAPG by strains 2P24 (pRK415), the *grxD* mutant (pRK415), the *grxD* mutant (p415-grxD), and the *grxD* mutant (p415-grxDM). **(B)** PG quantification from strains 2P24 (pRK415), the *grxD* mutant (pRK415), the *grxD* mutant (p415-grxD), and the *grxD* mutant (p415-grxDM) was measured by a colorimetric method. **(C)** The expression of *phlA* was positively regulated by GrxD. The β-galactosidase activity of the translational fusion *phlA*′-′*lacZ* was measured in strain 2P24 and its *grxD* mutant. **(D)** Inhibition of *Rhizoctonia solani* mycelial growth by 2P24 (pRK415), the *grxD* mutant (pRK415), the *grxD* mutant (p415-grxD), and the *grxD* mutant (p415-grxDM). All experiments were performed in triplicate, and letter represents significant differences between samples where *p* < 0.05.

The *grxD*, *grxC*, *grxF*, and *grxG* mutants were constructed to investigate the role of different GRXs on the production of 2,4-DAPG of *P. fluorescens*. HPLC indicated that the production of 2,4-DAPG in the *grxD* mutant was significantly impaired compared with that in strain 2P24 (Figure 1A). In addition, the production of PG, the intermediate of 2,4-DAPG, was severely decreased in the *grxD* mutant (Figure 1B). The complementation of *grxD* by introducing the complementary plasmid p415-grxD restored the 2,4-DAPG and PG production to wild-type levels (Figures 1A,B). In addition, the expression of the *phlA*′-′*lacZ* translational fusion on the plasmid p6013-phlA in the *grxD* mutant was significantly decreased compared with that in strain 2P24 (Figure 1C). GrxD contained the characterized monothiol glutaredoxin CGFS motif. To further determine whether this motif was required for GrxD-mediated 2,4-DAPG production in *P. fluorescens*, we generated a *grxD*^*C*29*S*^ variant by using site-directed mutagenesis, which converted the cysteine residue C29 into serine. The *grxD* mutant expressing GrxD^*C*29*S*^ was unable to restore 2,4-DAPG and PG production to those in strain 2P24, suggesting that the CGFS motif was necessary for the function of GrxD (Figures 1A,B). Furthermore, the *grxD* mutant failed to inhibit the growth of *R. solani*, whereas the *grxD* mutant expressing GrxD but not GrxD^*C*29*S*^ restored this inhibition to wild-type levels (Figure 1D). Interestingly, no differences in 2,4-DAPG production were observed among *grxC*, *grxF*, *grxG*, and the wild-type strain (Supplementary Figure 1). Considering that the *grx* genes belonging to the same class were functionally redundant, we then constructed the *grxC grxF* double mutant and the *grxC grxF grxG* triple mutant. Similarly, the *grxC grxF* double mutant and the *grxC grxF grxG* triple mutant produced similar levels of 2,4-DAPG as the wild-type strain 2P24 (Supplementary Figure 1). Furthermore, the quantification of acetyl-CoA (the substrate for PG biosynthesis) by using the acetyl-CoA assay kit showed no significant difference in the concentration of acetyl-CoA in strain 2P24 and its *grxD* mutant (Supplementary Figure 2). Overall, these results indicated that only *grxD* was specifically involved in the production of 2,4-DAPG in *P. fluorescens*.

### Deletion of *grxD* Enhances the RsmA/RsmE Protein Levels

**FIGURE 2:**
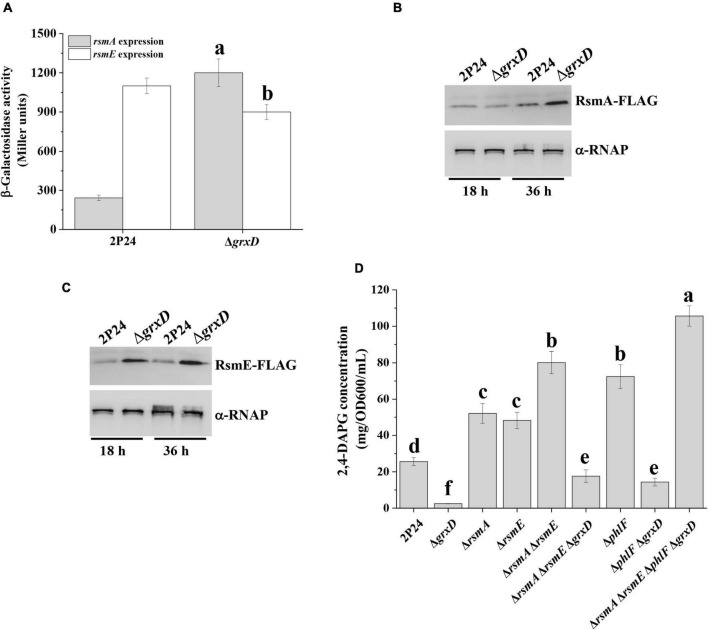
GrxD influenced the levels of RsmA and RsmE proteins. **(A)** The β-galactosidase activity of *rsmA*′-′*lacZ* and *rsmE*′-′*lacZ* translational fusions was determined in strain 2P24 and its *grxD* mutant at 36 h after inoculation. Analysis of RsmA-FLAG **(B)** or RsmE-FLAG **(C)** levels in total crude lysates prepared from strain 2P24 and its *grxD* mutant. An antibody directed against RNA polymerase β subunit (α-RNAP) was used as a loading control in this and later assays. Quantification of the production of 2,4-DAPG **(D)** from strain 2P24 and its derivatives. All experiments were performed in triplicate, and different letters indicate a significant difference at the 0.05 level.

In *P. fluorescens* 2P24, the Gac/Rsm signaling cascade plays a key role in controlling the production of 2,4-DAPG (Zhang et al., 2020b). Considering that the *grxD* mutant showed a 2,4-DAPG production phenotype similar to the *gacA* mutant (Figure 1), we speculated that the Gac/Rsm signaling cascade was involved in the positive role of 2,4-DAPG production by GrxD. To test this hypothesis, we initially assessed whether GrxD negatively regulated the expression of *rsmA* and *rsmE* in *P. fluorescens*. Translation fusion assays showed that the deletion of *grxD* significantly enhanced the *rsmA*′-′*lacZ* expression, whereas the *rsmE*′-′*lacZ* expression was slightly decreased in the *grxD* mutant compared with those in strain 2P24 (Figure 2A). To further gain insight into the effect of *grxD* on RsmA and RsmE, the protein levels of RsmA and RsmE were examined in the wild-type 2P24 and the *grxD* mutant. At 18 h after inoculation, the *grxD* mutant produced significantly enhanced levels of RsmE and similar levels of RsmA compared with the wild-type strain 2P24 (Figures 2B,C). However, at 36 h after inoculation, the levels of RsmA and RsmE were significantly increased in the *grxD* mutant (Figures 2B,C). Later, the effect of *grxD* on the transcription of *rsmZ*, *rsmY*, *rsmX*, *rsmX1*, and *rgsA* was then investigated. As shown in Supplementary Figure 2, the *grxD* mutant had unchanged expression levels of *rgsA*, *rsmX1*, *rsmY*, and *rsmZ*, but significantly increased expression of *rsmX* compared with the 2P24 strain (Supplementary Figure 3A). Our previous data showed that the lack of *rsmX* in 2P24 could not influence 2,4-DAPG production (Zhang et al., 2020a). Interestingly, the production of 2,4-DAPG in the *grxD rsmX* double mutant was significantly decreased compared with the *grxD* mutant (Supplementary Figure 3B), suggesting that the mutation of *grxD* could improve the expression of *rsmX*, which competitively binds to the RsmA/RsmE proteins and thus influence the function of the RsmA/RsmE proteins. Therefore, these results indicated that the RsmA/RsmE proteins played an important role in the GrxD-mediated 2,4-DAPG production in strain 2P24.

The overexpression of RsmA and RsmE resulted in decreased 2,4-DAPG production (Zhang et al., 2020b). Thus, we investigated the effect of RsmA and RsmE on GrxD-mediated 2,4-DAPG production. Results indicated that the *rsmA* and the *rsmE* mutants produced a significantly higher amount of 2,4-DAPG than strain 2P24 (Figure 2D). The deletion of *rsmA* and *rsmE* genes in the *grxD* mutant background partially restored the production of 2,4-DAPG to the wild-type levels (Figure 2D). In addition, the production of PG and 2,4-DAPG in the *grxD* mutant was unchanged at 18 h after inoculation and significantly decreased at 36 h after inoculation compared with that in the wild-type strain (Supplementary Figure 4). Our previous study showed that the production of exoprotease is negatively regulated by RsmA/E proteins (Wei et al., 2004a). To further examine whether GrxD controlled the production of exoprotease in strain 2P24, we compared the production of exoprotease in the wild-type strain and its derivatives. The deletion of the *grxD* gene in the wild-type background significantly influenced the production of exoprotease (Supplementary Figure 5A). By contrast, the production of exoprotease in the *rsmA rsmE* double mutant was significantly increased compared with that in the wild type. As expected, the lack of *grxD* in the *rsmA rsmE* double mutant could not influence exoprotease production, suggesting that GrxD affected the production of exoprotease through RsmA/E proteins (Supplementary Figure 5A). A similar regulation was observed when the *lacZ*-fused translational reporter of *aprA* (exoprotease biosynthetic gene) was determined in 2P24 and its derivatives (Supplementary Figure 5B). Our results indicated that GrxD repressed the levels of RsmA and RsmE, which contributed, at least partially, to the positive regulatory effect of GrxD on the 2,4-DAPG production in strain 2P24.

### GrxD Regulates 2,4-Diacetylphloroglucinol Production Through the Transcriptional Regulator PhlF

**FIGURE 3:**
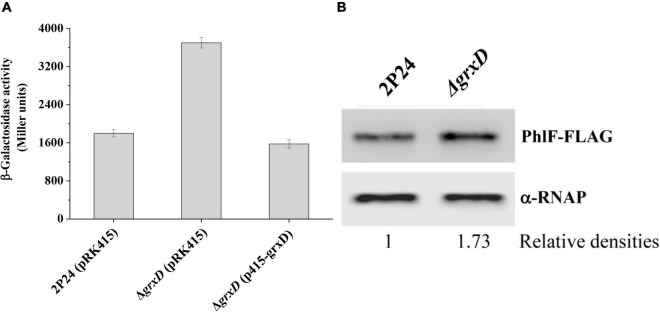
GrxD influenced *phlF* in *Pseudomonas fluorescens*. **(A)** The β-galactosidase activity of *phlF*-*lacZ* transcriptional fusion was determined in strains 2P24 (pRK415), the *grxD* mutant (pRK415), and the *grxD* mutant (p415-grxD). **(B)** Western blot analysis was performed to detect the level of PhlF-FLAG. The relative intensities of PhlF-FLAG bands were marked below the blot. All experiments were performed in triplicate, and different letters indicate a significant difference at the 0.05 level.

To further investigate how GrxD influences 2,4-DAPG production, we attempted to identify potential target genes regulated by GrxD. Previous work demonstrated that the expression of *phlACBD* was negatively regulated by the pathway-specific repressor PhlF (Abbs et al., 2002; Zhou et al., 2010). We hypothesized that GrxD negatively influenced *phlF* and then positively affected the production of 2,4-DAPG in *P. fluorescens*. To test this hypothesis, we assessed the expression of *phlF* in the wild-type strain and the *grxD* mutant. The deletion of *grxD* significantly increased *phlF* expression, and the complementation of the *grxD* mutant restored *phlF* expression to wild-type levels (Figure 3A). We then monitored the levels of the PhlF protein in strain 2P24 and its *grxD* mutant by Western blotting. In agreement with the *phlF* transcriptional levels, the Western blot assay showed that levels of the PhlF protein in the *grxD* mutant were increased compared with that of 2P24 (Figure 3B). These data supported our hypothesis and suggested that GrxD negatively controlled the expression of *phlF*.

Later, we constructed the *grxD phlF* double mutant and compared its 2,4-DAPG production to that of the wild-type strain 2P24 and its derivatives. Our data indicated that the production of 2,4-DAPG in the *grxD phlF* double mutant was higher compared with that in the *grxD* mutant but was significantly lower compared with the wild-type strain (Figure 2D). Furthermore, the *rsmA rsmE phlF grxD* quadruple mutant was constructed, and HPLC showed that the production of 2,4-DAPG in the *rsmA rsmE phlF grxD* quadruple mutant was significantly increased compared with that in the *phlF* mutant or the *rsmA rsmE* double mutant (Figure 2D). These results suggested that the transcriptional regulator PhlF and RsmA/E proteins were involved in the GrxD-mediated regulation of 2,4-DAPG.

### GrxD Influences the Growth of *Pseudomonas fluorescens*

**FIGURE 4:**
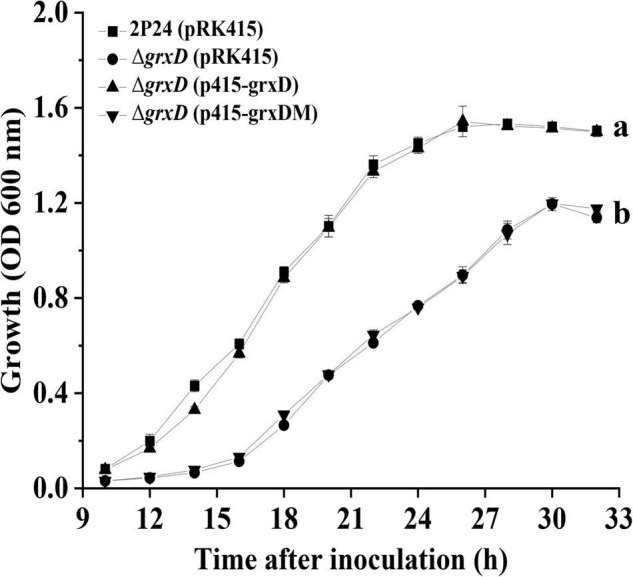
The effect of *grxD* gene on the growth of *Pseudomonas fluorescens*. Strain 2P24 and its derivatives were cultured in ABM medium and the absorbance at 600 nm were measured at different points of the growth curve. All experiments were performed in triplicate, and different letters indicate a significant difference at the 0.05 level.

Given the imbalance of redox state in *grx* mutants and the involvement of GRXs in regulating bacterial growth (Cao et al., 2020), the growth of all *grx* mutants was measured in an ABM medium. Our data indicated that the *grxD* mutant showed a significantly lower than the wild-type strain in the ABM minimal medium (Figure 4). This growth defect could be restored to wild-type levels by complementation with p415-grxD but not p415-grxDM (Figure 4). By contrast, no difference could be observed among the *grxC*, *grxF*, and *grxG* mutants, and strain 2P24 in the ABM medium. Furthermore, the *grxC grxF* double mutant and the *grxC grxF grxG* triple mutant were indistinguishable from the wild-type strain (Supplementary Figure 6). Thus, the monothiol glutaredoxin GrxD was important for the normal growth of *P. fluorescens*.

### *grxD* Mutant Is Sensitive Under Oxidative Stress Conditions

**FIGURE 5:**
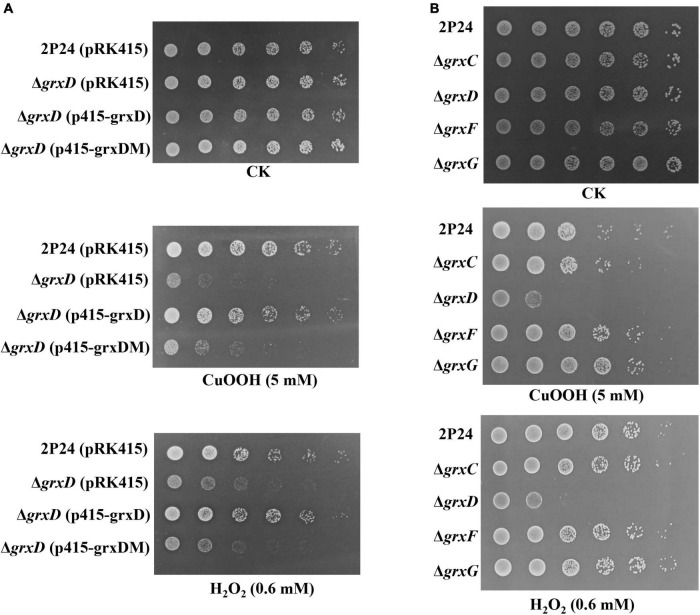
Effect of the *grx* mutants on tolerance of *Pseudomonas fluorescens* to peroxides. *Pseudomonas fluorescens* 2P24 (pRK415), the *grxD* mutant (pRK415), the *grxD* mutant (p415-grxD), and the *grxD* mutant (p415-grxDM) **(A)**, or 2P24, the *grxC* mutant, the *grxD* mutant, the *grxF* mutant, and the *grxG* mutant **(B)** were grown in Lysogeny Broth (LB) broth, serially diluted and spotted onto ABM plates or ABM plates containing 5 mM CuOOH or 0.6 mM H_2_O_2_. Plates were incubated at 28°C for 2 days.

The survival of strain 2P24 and its derivatives was examined *via* killing assays. As shown in Figure 5, the *grxD* mutant showed significantly increased susceptibility to relatively high H_2_O_2_ (0.6 mM) or CuOOH (5 mM). The complementation of the *grxD* mutant by expression of *grxD* in trans restored H_2_O_2_ or CuOOH resistance to that of the wild-type strain, whereas the *grxD* mutant expressing GrxD^*C*29*S*^ exhibited the same phenotype of the *grxD* mutant (Figure 5A). In addition, the *grxC*, *grxF*, or *grxG* mutant was as sensitive as the wild-type strain to H_2_O_2_ or CuOOH (Figure 5B), suggesting that the monothiol GRX GrxD played an important role in the resistance of *P. fluorescens* against oxidative stress.

### Mutation of the *grxD* Gene Decreases the Production of Siderophores in *Pseudomonas fluorescens*

**FIGURE 6:**
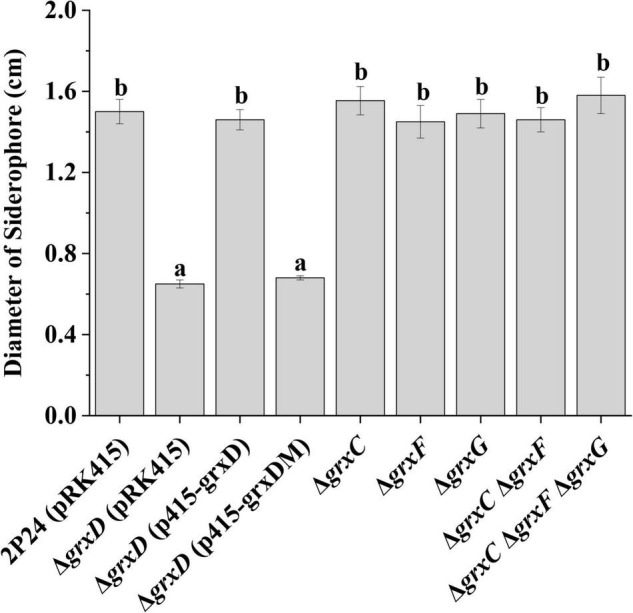
Effects of *grx* mutants on siderophore production of *Pseudomonas fluorescens*. Siderophore production of strain 2P24 and its derivatives was indicated by the diameter of the orange circle on the chrome azurol S (CAS) plate. The experiment was performed in triplicate, and different letters indicate a significant difference at the 0.05 level.

To determine whether GrxD influenced iron availability in the environment, we compared the production of siderophores in the wild-type strain and its *grx* mutants. The siderophore activity was analyzed using the CAS assay. The *grxD* mutant showed a significantly decreased amount of siderophores, which was restored in the complemented strain (Figure 6). By contrast, the amount of siderophores produced by the *grxC*, *grxF*, *grxG* mutants, the *grxC grxF* double mutant, or the *grxC grxF grxG* triple mutant was indistinguishable from that produced by the wild-type strain (Figure 6). These results demonstrated the important role of GrxD in the production of siderophores of *P. fluorescens*.

### Effects of *grx* Mutants on the Concentration of Iron

**FIGURE 7:**
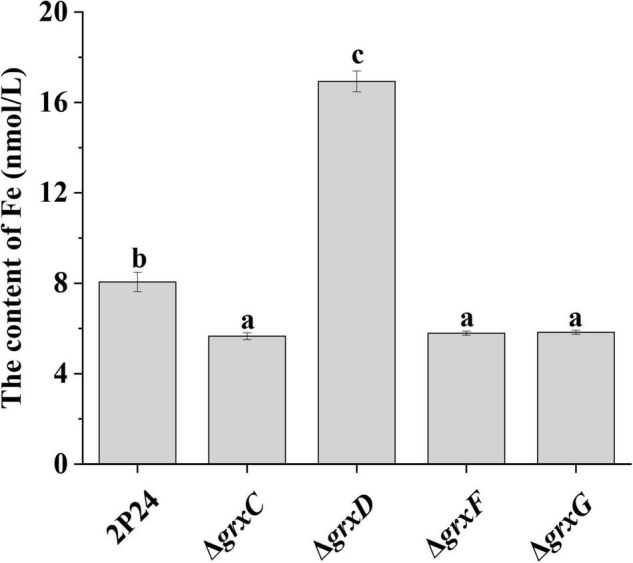
Deletion of *grx* genes influenced the iron content of *Pseudomonas fluorescens*. The Fe content was measured in 3 × 10^9^ cells of strain 2P24 and its derivatives using the ferrozine assay. The experiment was performed in triplicate, and different letters indicate a significant difference at the 0.05 level.

To assess the effect of *grx* genes on the accumulation of iron in *P. fluorescens* cells, we then measured the intracellular Fe content in 2P24 and its derivatives. The Fe content in the *grxC*, *grxF*, and *grxG* mutants was significantly lower than that in the 2P24 strain (Figure 7). By contrast, the deletion of the monothiol *grxD* gene resulted in a dramatic accumulation of total Fe content in the cells of *P. fluorescens* (Figure 7). These results suggested that the monothiol GrxD played an important role in the Fe homeostasis of *P. fluorescens*.

### Effect of the *grx* Mutation on the Motility of *Pseudomonas fluorescens*

**FIGURE 8:**
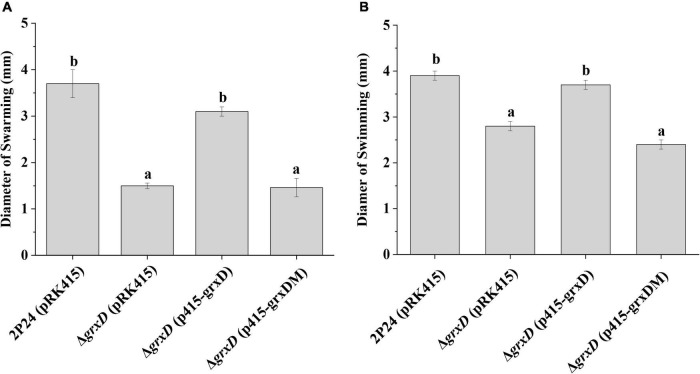
Effect of the *grxD* gene on the motility of *Pseudomonas fluorescens*. **(A)** Swarming agar plate assay. The swarming abilities of strain 2P24 and its derivatives were determined by the diameter of the zone of motility. **(B)** Swimming agar plate assay. The swimming abilities of strain 2P24 and its derivatives were determined by the diameter of the zone of motility. The experiments were performed in triplicate, and different letters indicate a significant difference at the 0.05 level.

Motility is important for the establishment of the bacteria in the rhizosphere and bacterial performance as PGPR (Barret et al., 2011). The effect of the *grx* mutants on motility was then measured. Compared with the wild-type strain, the loss of the GrxD function affected the swarming or swimming motility (Figure 8). This observed phenotype in the *grxD* mutant could be complemented by introducing a copy of wild-type *grxD*. Furthermore, the characterized active site (CGFS) motif of GrxD played a vital role in bacterial motility (Figure 8). However, no such difference in swarming or swimming motility could be observed between the wild-type strain and the *grxC*, *grxF*, and *grxG* mutants, the *grxC grxF* double mutant, or the *grxC grxF grxG* triple mutant (data not shown). These results indicated that GrxD positively affected motility in *P. fluorescens*.

### GrxD Is Required for the Biocontrol Capacity of *Pseudomonas fluorescens*

GrxD positively affects the production of 2,4-DAPG, which is essential for protecting tomatoes against *R. solanacearum*. To assess the relative contribution of *grx* genes to the biocontrol capacity of *P. fluorescens*, we then investigated the effect of strain 2P24 and its derivatives in controlling the bacterial wilt of tomatoes. The *grxD* mutant was significantly impaired in its capacity to protect tomatoes from root diseases (Table 1). The biocontrol activity of the *grxD* mutant could be restored to the wild-type level by complementing the *grxD* mutant with p415-grxD, but not p451-grxDM. The deletion of *grxC*, *grxF*, or *grxG* in 2P24 did not result in a significant alteration in biocontrol efficacy (Table 1). These results indicated that GrxD played an important role in the biocontrol capacity of *P. fluorescens*.

**TABLE 1:** Contribution of *grx* genes in strain 2P24 to the suppression of tomato bacterial wilt caused by Ralstonia solanacearum*.

Treatment	Disease index		Surviving plants per flask (%)	
	
	7 days	14 days	7 days	14 days
None	26.1a	68.9a		
2P24 (pRK415)	8.3c	26.1c	68.7	67.1
Δ*grxC* (pRK415)	9.9c	26.1c	68.5	60.8
Δ*grxF* (pRK415)	8.9c	26.1c	68.1	66.3
Δ*grxG* (pRK415)	10c	26.7c	67.7	61.5
Δ*grxD* (pRK415)	16.7b	53.3b	56.1	35.4
Δ*grxD* (p415-grxD)	8.9c	31.2c	66.3	61.9
Δ*grxD* (p415-grxDM)	12.8b	53.4b	54.5	34.9

*Data are averages of three repeats and means within the same column by different letters differ significantly at P = 0.05, according the Student’s t-test.

## Discussion

Glutaredoxin is an antioxidant protein that plays an important role in various physiological processes, such as stress response, petal development, Fe–S cluster assembly, signaling and pathogen response in bacteria, fungi, and mammals (Couturier et al., 2015). The results of this study have advanced our knowledge in the functions of glutaredoxin and have shown that monothiol GRX GrxD plays roles in many important biocontrol-related traits, such as the production of the antibiotic 2,4-DAPG, siderophores, motility, and biocontrol efficacy of the plant beneficial bacterium *P. fluorescens* 2P24.

Previous work showed that 2,4-DAPG-producing pseudomonads have evolved sophisticated mechanisms to fine-tune the concentration of 2,4-DAPG in the cells. Among these mechanisms is the Gac/Rsm signal cascade. This cascade involves the GacS/GacA two-component system, which induces the expression of sRNAs, and the RNA-binding proteins RsmA and RsmE (Lapouge et al., 2008). RsmA and RsmE are predicted to bind to the ribosome-binding site of *phlACBD* mRNA directly, thereby repressing its translation and influencing the production of 2,4-DAPG (Zhang et al., 2020b). To date, some genetic elements that regulate the expression of sRNAs have been described in detail. In *P. aeruginosa*, the expression levels of *rsmZ* are negatively regulated by the phosphotransfer protein HptB and H-NS family member MvaT (Brencic et al., 2009). In addition, IHF, PsrA, and AlgR are involved in inducing the expression of *rsmZ* of *P. protegens* CHA0 (Humair et al., 2010). Compared with the well-studied regulation of these sRNAs, few regulators are reported to have regulatory effects on the expression of their cognate small regulatory proteins RsmA or RsmE. Our previous work showed that c-di-GMP positively regulates the expression levels of *rsmA* and *rsmE* in strain 2P24 (Liang et al., 2020). Our findings here showed that GrxD controls the expression of RsmA and RsmE (Figure 2). However, the regulatory effects of GrxD on *rsmA* and *rsmE* are unique. Unlike c-di-GMP, GrxD could influence the levels of RsmA protein at the stationary phase (36 h), whereas RsmE was significantly altered during the exponential (18 h) and stationary (36 h) phases (Figure 2). Regarding the complex effect of RsmA and RsmE on 2,4-DAPG, this work suggested that the effect of 2,4-DAPG by GrxD is achieved through fine-tuning the protein levels of RsmA and RsmE in the cells.

Besides the RsmA and RsmE proteins, our data revealed that GrxD positively influenced the production of 2,4-DAPG by repressing the expression of *phlF* (Figure 3). PhlF is a transcriptional regulator for the 2,4-DAPG biosynthetic operon. Sequence analysis reveals that PhlF is a cytoplasmic protein with four cysteine residues (data not shown). Considering that the GRX can influence the disulfide bonds formed between cysteine residues of target proteins and glutathione, we hypothesize that GrxD directly interacts with PhlF. However, the bacterial two-hybrid assay shows no interaction between GrxD and PhlF *in vivo* (data not shown). Previous studies indicated that GrxD could directly interact with the iron-responsive transcriptional regulators Aft1 and Aft2 in *Saccharomyces cerevisiae* (Ojeda et al., 2006). Aft1 induces the expression of RNA-binding protein CTH2, which degrades the transcripts of iron-requiring enzymes to conserve iron in the cell. Since the mutation of *grxD* could induce the accumulation of iron in *P. fluorescens* 2P24. Homologs of CTH2, such as C0J56_01730, C0J56_04495, and C0J56_06345, can be found in strain 2P24. It is possible that the iron-responsive transcriptional factors could be activated in the *grxD* mutant to induce these RNA-binding proteins, which could inhibit the translation of RsmA/E proteins. Furthermore, the mutation of *grxD* could affect the enzyme activity of PhlA, PhlC, PhlB, or PhlD. Thus, the mechanism behind GrxD in the production of 2,4-DPAG remains to be elucidated.

The characterization of *grx* mutants indicates that monothiol GRX is involved in influencing cellular redox state, and this finding is in agreement with phenotypes previously described in *Azorhizobium caulinodans* and *Rhizobium leguminosarum* bv. *viciae* (Cao et al., 2020; Zou et al., 2021). In *P. fluorescens*, the mutation of *grxD* is hypersensitive to H_2_O_2_ and CuOOH, whereas the *grxC*, *grxF*, *grxG* mutants, and the *grxC grxF grxG* triple mutant display no such effect (Figure 5). Monothiol GRX is essential for the Fe–S cluster biosynthesis, and our data also indicate that GrxD regulates siderophore production and is involved in the iron homeostasis of *P. fluorescens* (Figures 6, 7). Overall, our data have characterized the essential role of GrxD of *P. fluorescens* in oxidative stress.

**FIGURE 9:**
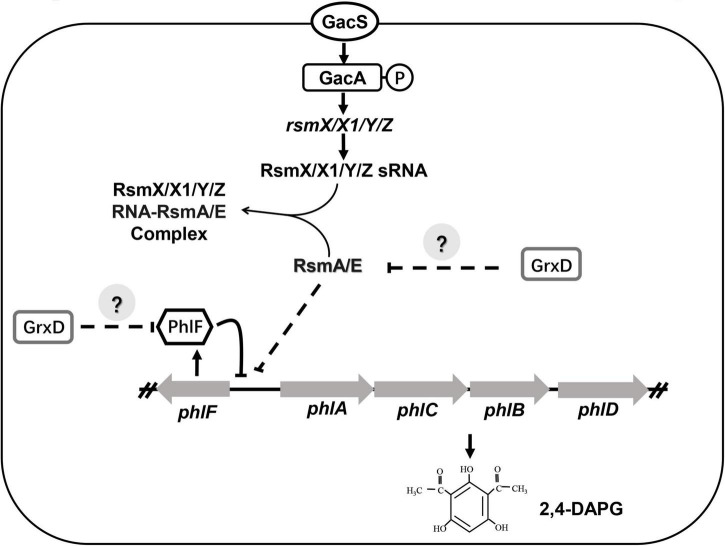
Model of GrxD affects the production of 2,4-DAPG in *Pseudomonas fluorescens* 3P24. The production of 2,4-diacetylphloroglucinol (2,4-DAPG) was previously reported to be regulated by the Gac/Rsm signaling pathway and the transcriptional factor PhlF. In this study, the levels of RsmA/E and PhlF proteins were modulated by GrxD. In addition, GrxD could interact directly with an unknown transcriptional regulator, which may influence the expression of *rsmA/E* and *phlF*.

In plant assays carried out under greenhouse conditions, the mutation of *grxD* severely impairs the biocontrol activity against tomato bacterial wilt caused by *R. solanacearum* (Table 1). By contrast, the *grxC*, *grxF*, and *grxG* mutants provide a similar effect in protecting tomatoes from tomato bacterial wilt as the wild-type strain. Our previous data suggested that 2,4-DAPG plays a vital role in the biocontrol activity of *P. fluorescens* (Table 1). Given the effect of GrxD on many biocontrol traits, such as motility, bacterial resistance against peroxides, and the production of siderophore, defects in biocontrol capacity can be a combined result. Therefore, our work indicates that GrxD plays a vital role in the regulation of the biocontrol ability and is necessary for the ecological fitness of *P. fluorescens* in a constantly changing environment (Figure 9).

## Data Availability Statement

The raw data supporting the conclusions of this article will be made available by the authors, without undue reservation.

## Author Contributions

XW conceived and designed the study. QD, QY, BZ, and XW performed the assays. QD, QY, and BZ collected the data. L-QZ and XW analyzed the data and wrote the manuscript. All authors contributed to the article and approved the submitted version.

## Conflict of Interest

The authors declare that the research was conducted in the absence of any commercial or financial relationships that could be construed as a potential conflict of interest.

## Publisher’s Note

All claims expressed in this article are solely those of the authors and do not necessarily represent those of their affiliated organizations, or those of the publisher, the editors and the reviewers. Any product that may be evaluated in this article, or claim that may be made by its manufacturer, is not guaranteed or endorsed by the publisher.
