# Supplementary material for: Effect of the Monothiol Glutaredoxin GrxD on 2,4-Diacetylphloroglucinol Biosynthesis and Biocontrol Activity of Pseudomonas fluorescens 2P24
Source: Front Microbiol. 2022 Jul 8;13:920793. doi: 10.3389/fmicb.2022.920793 (PMC9304865; doi:10.3389/fmicb.2022.920793)
Supplement: Supplementary file 1 [file Data_Sheet_1.doc]

**Supporting information**

**Table S1 Bacterial strains and plasmids used in this study**

| **Strains or plasmids** | **Relevant characteristics*^a^*** | **Reference or source** |
| --- | --- | --- |
| **Strains** |  |  |
| *Pseudomonas fluorescens* |  |  |
| 2P24 | Wild-type, Ap^r^ | Wei et al., 2004 |
| WPM12 | Double deletion of *rsmA* and *rsmE*, Ap^r^ | Zhang et al., 2020a |
| PM911 | In-frame deletion of *phlF*, Ap^r^ | Zhou et al., 2010 |
| WPM31 | In-frame deletion of *grxD*, Ap^r^ | This work |
| WPM32 | In-frame deletion of *grxC*, Ap^r^ | This work |
| WPM33 | In-frame deletion of *grxF*, Ap^r^ | This work |
| WPM34 | In-frame deletion of *grxG*, Ap^r^ | This work |
| WPM35 | Double deletion of *grxC* and *grxF*, Ap^r^ | This work |
| WPM36 | Triple deletion of *grxC*, *grxF*, and *grxG*, Ap^r^ | This work |
| WPM37 | Triple deletion of *rsmA,* *rsmE*, and *grxD*, Ap^r^ | This work |
| WPM38 | Double deletion of *phlF* and *grxD*, Ap^r^ | This work |
| WPM39 | Double deletion of *rsmX* and *grxD*, Ap^r^ | This work |
| WPM40 | Quadruple deletion of *phlF*, *rsmA,* *rsmE*, and *grxD*, Ap^r^ | This work |
| WPM26 | Strain 2P24 with a FLAG epitope sequence tagged to the C terminus of RsmA, Ap^r^ | Liang et al., 2020 |
| WPM27 | Strain 2P24 with a FLAG epitope sequence tagged to the C terminus of RsmE, Ap^r^ | Liang et al., 2020 |
| WPM41 | Strain WPM31 with a FLAG epitope sequence tagged to the C terminus of RsmE, Ap^r^ | This work |
| WPM42 | Strain 2P24 with a FLAG epitope sequence tagged to the C terminus of PhlF, Ap^r^ | Lab stock |
| WPM43 | Strain WPM28 with a FLAG epitope sequence tagged to the C terminus of PhlF, Ap^r^ | This work |
| *Rhizoctonia solani* | Basidiomycete fungus caused cotton blight | Lab stock |
| *Ralstonia solanacearum* | Bacterial pathogen caused tomato bacterial wilt | Lab stock |
| *E. coli* DH5α | *supE44* *lacU*169 (*φ80lacZ* M15) *hsdR*17 *recA*1 *endA*1 *gyrA*96 *thi*-1 *relA*1 | Sambrook et al., 1989 |
|  |  |  |
| **Plasmids** |  |  |
| pRK415 | Broad-host-range cloning vector, Tet^r^ | Keen et al., 1988 |
| p415-grxD | pRK415 containing the *grxD* gene, Tet^r^ | This work |
| p415R-grxDM | p415-grxD derivative with CGFS to SGFS point mutation, Tet^r^ | This work |
| pBBR1MCS-2 | Broad-host-range cloning vector, Km^r^ | Kovach et al., 1995 |
| pBBR-icsA | pBBR1MCS-2 containing the *icsA* gene, Km^r^ | This work |
| p970Gm-rsmXp | *rsmX*-*lacZ* transcriptional fusion, Gm^r^ | Zhang et al., 2020a |
| p970Gm-rsmX1p | *rsmX1*-*lacZ* transcriptional fusion, Gm^r^ | Zhang et al., 2020a |
| p970Gm-rsmYp | *rsmY*-*lacZ* transcriptional fusion, Gm^r^ | Zhang et al., 2020a |
| p970Gm-rsmZp | *rsmZ*-*lacZ* transcriptional fusion, Gm^r^ | Zhang et al., 2020a |
| p970Gm-rgsAp | *rgsA*-*lacZ* transcriptional fusion, Gm^r^ | Zhang et al., 2020a |
| p970Km-phlFp | *phlF*-*lacZ* transcriptional fusion, Km^r^ | Zhang et al., 2020b |
| p6013-rsmAp | *rsmA′*-*′lacZ* translational fusion, Tet^r^ | Zhang et al., 2020b |
| p6013-rsmEp | *rsmE′*-*′lacZ* translational fusion, Tet^r^ | Zhang et al., 2020b |
| p6013-phlAp | *phlA′*-*′lacZ* translational fusion, Tet^r^ | Zhang et al., 2020b |
| p6014-aprAp | *aprA′*-*′lacZ* translational fusion, Tet^r^ | Zhang et al., 2020b |
| p2P24Km | Sucrose-based counter-selectable plasmid, Km^r^ | Zhang et al., 2018 |
| p2P24-grxD | p2P24Km carrying a deleted *grxD* gene, Km^r^ | This work |
| p2P24-grxC | p2P24Km carrying a deleted *grxC* gene, Km^r^ | This work |
| p2P24-grxF | p2P24Km carrying a deleted *grxF* gene, Km^r^ | This work |
| p2P24-grxG | p2P24Km carrying a deleted *grxG* gene, Km^r^ | This work |

*^a^*Ap, ampicillin; Gm, gentamicin; Km, kanamycin; Tet, tetracycline.

**References**

Keen, N.T., Tamaki, S., Kobayashi, D., Trollinger, D., 1988. Improved broad-host-range plasmids for DNA cloning in gram-negative bacteria. Gene 70, 191-197.

Kovach, M.E., Elzer, P.H., Hill, D.S., Robertson, G.T., Farris, M.A., Roop, R.M., Peterson, K.M., 1995. Four new derivatives of the broad-host-range cloning vector pBBR1MCS, carrying different antibiotic-resistance cassettes. Gene 166, 175-176.

Liang, F., Zhang, B., Yang, Q., Zhang, Y., Zheng, D., Zhang, L.Q., Yan, Q., Wu, X., 2020. Cyclic-di-GMP regulates the quorum-sensing system and biocontrol activity of *Pseudomonas fluorescens* 2P24 through the RsmA and RsmE proteins. Appl. Environ. Microbiol. 86, e02016-20.

Sambrook, J., Fritsch, E.F., Maniatis, T., 1989. Molecular cloning: a laboratory manual, 2nd ed. Cold Spring Harbor Laboratory Press, Cold Spring Harbor, N. Y.

Wei, H.L., Wang, Y., Zhang, L.Q., Tang, W.H., 2004. Identification and characterization of biocontrol bacterial strain 2P24 and CPF-10. Acta Phytopathol. Sin. 34, 80-85.

Zhang, Y., Zhang, B., Wu, X.G., Zhang, L.Q., 2020a. Characterization the role of GacA-dependent small RNAs and RsmA family proteins on 2,4-diacetylphloroglucinol production in *Pseudomonas* *fluorescens* 2P24. Microbiol. Res. 233, 126391.

Zhang, Y., Zhang, B., Wu, H., Wu, X., Yan, Q., Zhang, L.Q., 2020b. Pleiotropic effects of RsmA and RsmE proteins in *Pseudomonas* *fluorescens* 2P24. BMC Microiol. 20, 191.

Zhang, Y., Zhang, Y., Zhang, B., Wu, X., Zhang, L.Q., 2018. Effect of carbon sources on production of 2,4-diacetylphloroglucinol in *Pseudomonas* *fluorescens* 2P24. Acta Microbiol. Sin. 58, 1202-1212.

Zhou, Y.P., Wu, X.G., Zhou, H.Y., He, Y.Q., Zhang, L.Q., 2010. Effect of gene *phlF* on 2,4-diacetylphloroglucinol production in *Pseudomonas* *fluorescens* 2P24. Acta Phytopathol. Sin. 40, 144-150.

**Table S2 Primers used in this study**

| **Name** | **Sequence (5′-3′)***^a^* | **Comment** |
| --- | --- | --- |
| grxD-EcoRI-F1 | CCGAATTCCTGTTCCTTGAAGGCCT | Constructing of in-frame *grxD* deletion mutant |
| grxD-R1-600bp | CCGTCCTGCTTTACATGAAAGGCTCAGCACGATCAAGCAAGCGGTCGAAA |
| grxD-F2-670bp | TTTCGACCGCTTGCTTGATCGTGCTGAGCCTTTCATGTAAAGCAGGACGG |
| grxD-BamHI-R2 | CAGGATCCACGCTTGAAAACCGAAA |
| grxG-F1-SacI | ATGAGCTCAAGCGCACCATGATGGAAGGCCGCGAC | Constructing of in-frame *grxG* deletion mutant |
| grxG-R1 | CATCCAGGAGGTCTGGCCATTCTCTTCGATGAAGATGATGAGCTGGCCCA |
| grxG-F2 | TGGGCCAGCTCATCATCTTCATCGAAGAGAATGGCCAGACCTCCTGGATG |
| grxG-R2-BamHI | ATGGATCCTGATCGAGGACGAGCCCTACCGCGAAC |
| grxC-EcoRI-F1 | ATGAATTCGGAGTTGCCCATCTGCCCGTC | Constructing of in-frame *grxC* deletion mutant |
| grxC-R1-780bp | CATCGAGCTTGCCGGCGCGCTCCAGGTAATCGCTGGAATAGACAACGACG |
| grxC-F2-880bp | CGTCGTTGTCTATTCCAGCGATTACCTGGAGCGCGCCGGCAAGCTCGATG |
| grxC-BamHI-R2 | ATGGATCCTGTTCGGCCCGGAAAGCCGTG |
| grxF-EcoRI-F1 | ATGAATTCAGCGGCTGGCTGAAGGAATACCTG | Constructing of in-frame *grxF* deletion mutant |
| grxF-R1-810bp | CGCGGACAACGCCTTGAGAATATTCAACTTTTTCAGTACCCTGCCGAGCA |
| grxF-F2-760bp | TGCTCGGCAGGGTACTGAAAAAGTTGAATATTCTCAAGGCGTTGTCCGCG |
| grxF-XbaI-R2 | ATTCTAGACCGCGCCGGGCTGTTCAGCGGTG |
| iscA-XbaI-F1 | ATTCTAGATTACGCCGCGCAGAAAG | Cloning the *iscA* gene |
| iscA-SacI-R2 | ATGAGCTCTTCGAGCTGGCGCACTT |
| grxD-F1-EcoRI | CCGAATTCCTGTTCCTTGAAGGCCT | Cloning the *grxD* gene |
| grxD-R2-BamHI | CAGGATCCACGCTTGAAAACCGAAA |
| grxD-C29S-R1-600bp | CGAACGCGCC ACAGTTCGGT TTCTCGGC | Site-directed mutagenesis of the active site (CGFS) motif of GrxD |
| GrxD-C29S-F2-670bp | GAACTGTGGC GCGTTCGGCG AGCCTTTC |  |

*^a^*Restriction sites inserted in the primer for the cloning strategy are underlined.

**Table S3 Antifungal activity of 2P24 and its derivatives against *R. solani* on PDA plates**

| **Name** | **Zone of suppression in PDA plates (mm)** |
| --- | --- |
| 2P24 | 4.1 ± 0.2 |
| 2P24M-05 | 2.5 ± 0.2b |
| 2P24M-12 | 2.6 ± 0.8b |
| 2P24M-39 | 0d |
| 2P24M-159 | 1.7 ± 0.4c |
| 2P24M-567 | 1.3 ± 0.6c |
| 2P24M-1230 | 3.1 ± 0.5a |

* The experiment was performed in triplicate, and different letters indicate a significant difference at the 0.05 level.

**
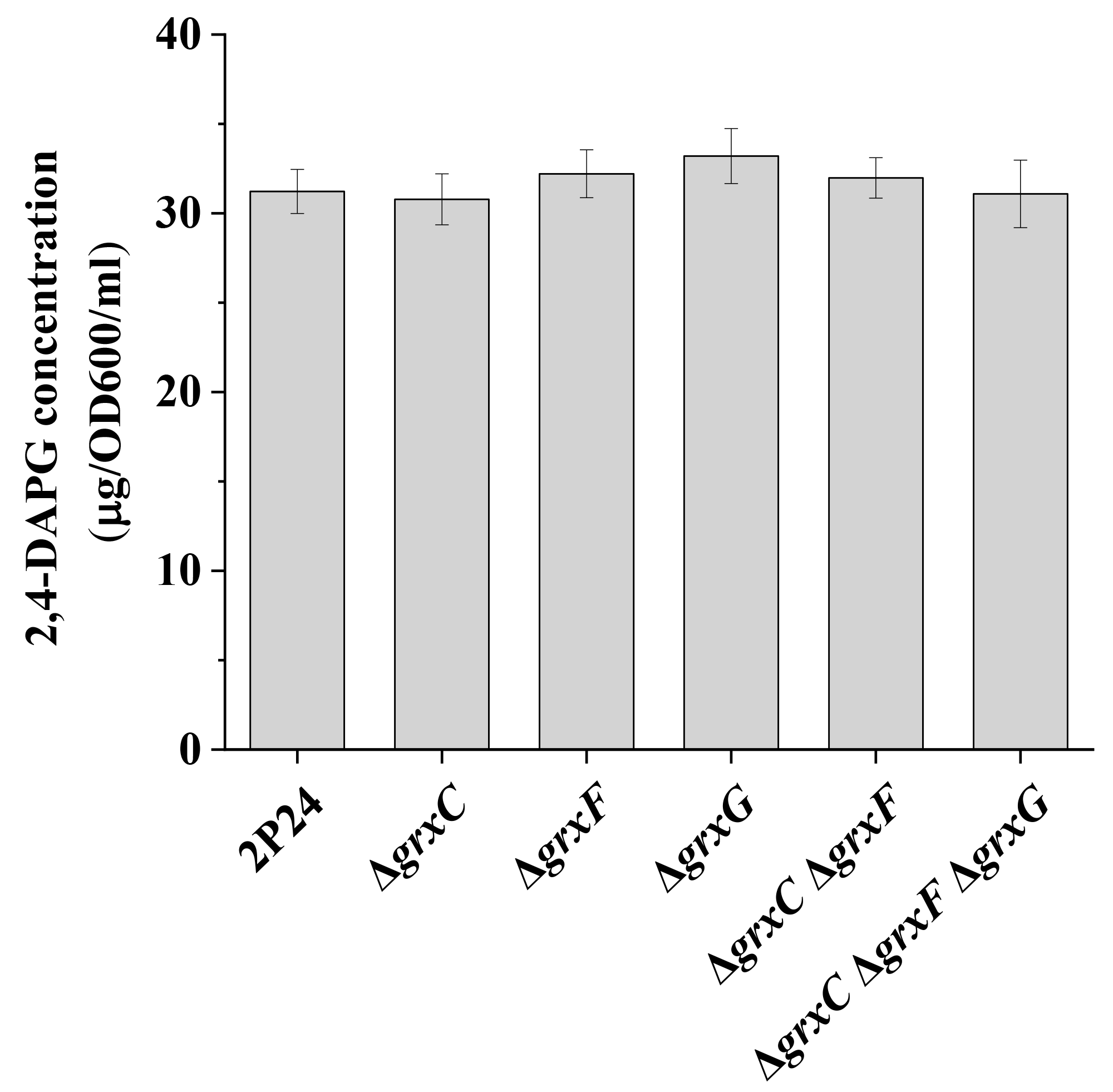
**

Fig. S1. The effect of *grx* genes on 2,4-DAPG biosynthesis of *P. fluorescens* 2P24. HPLC analysis of the concentration of 2,4-DAPG by strains 2P24, the *grxC* mutant, the *grxF* mutant, the *grxG* mutant, the *grxC* *grxF* double mutant, and the *grxC* *grxF* *grxG* triple mutant. All experiments were performed in triplicate, and the mean values ± standard deviations are indicated, **P* < 0.05.


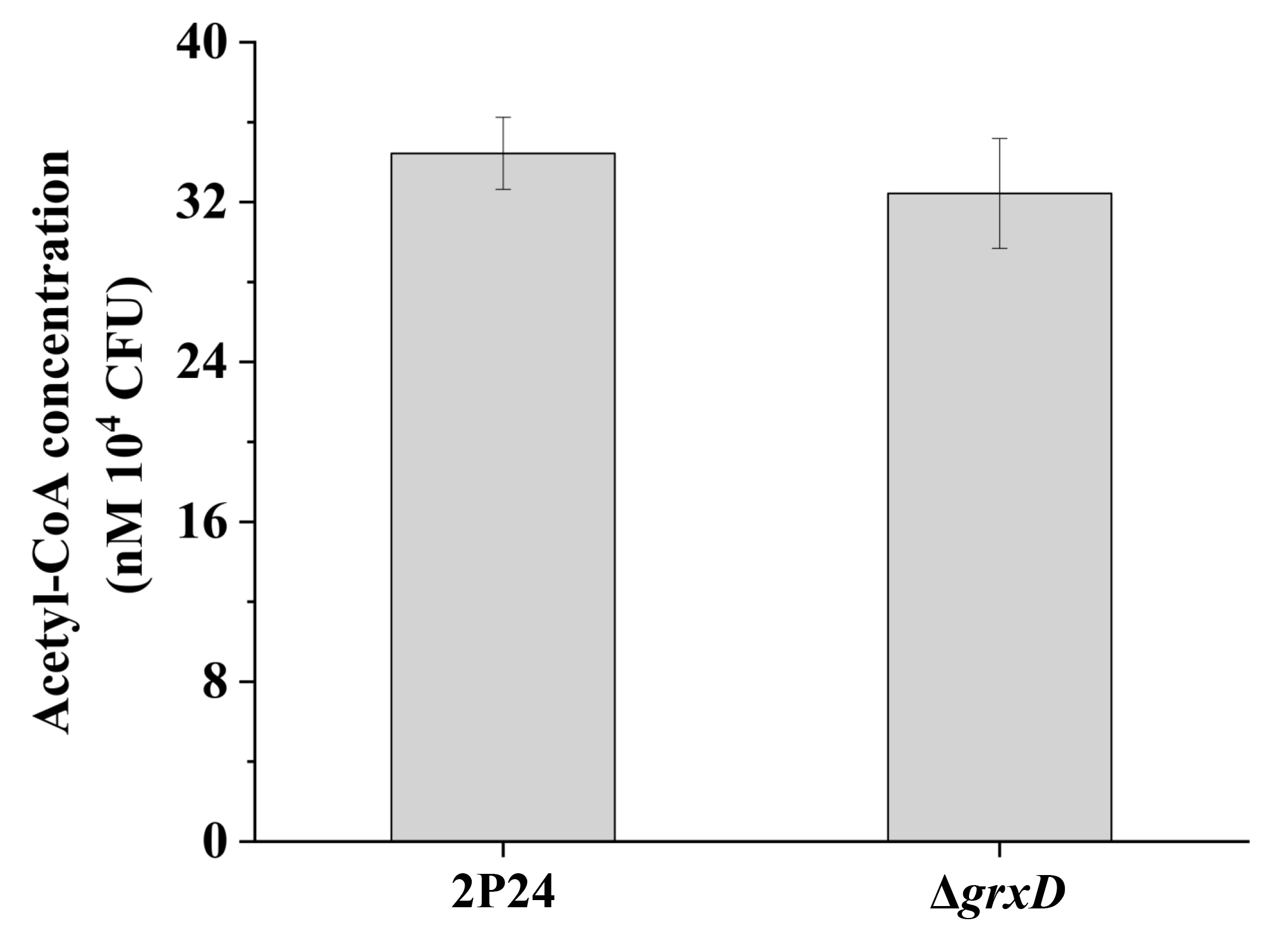
Fig. S2. The effect of *grxD* gene on the concentration of acetyl-CoA of *P. fluorescens* 2P24. Intracellular acetyl-CoA concentrations of strain 2P24 and its *grxD* mutant were determined using the PicoProbe acetyl-CoA assay kit (BioVision). All experiments were performed in triplicate, and the mean values ± standard deviations are indicated, **P* < 0.05.


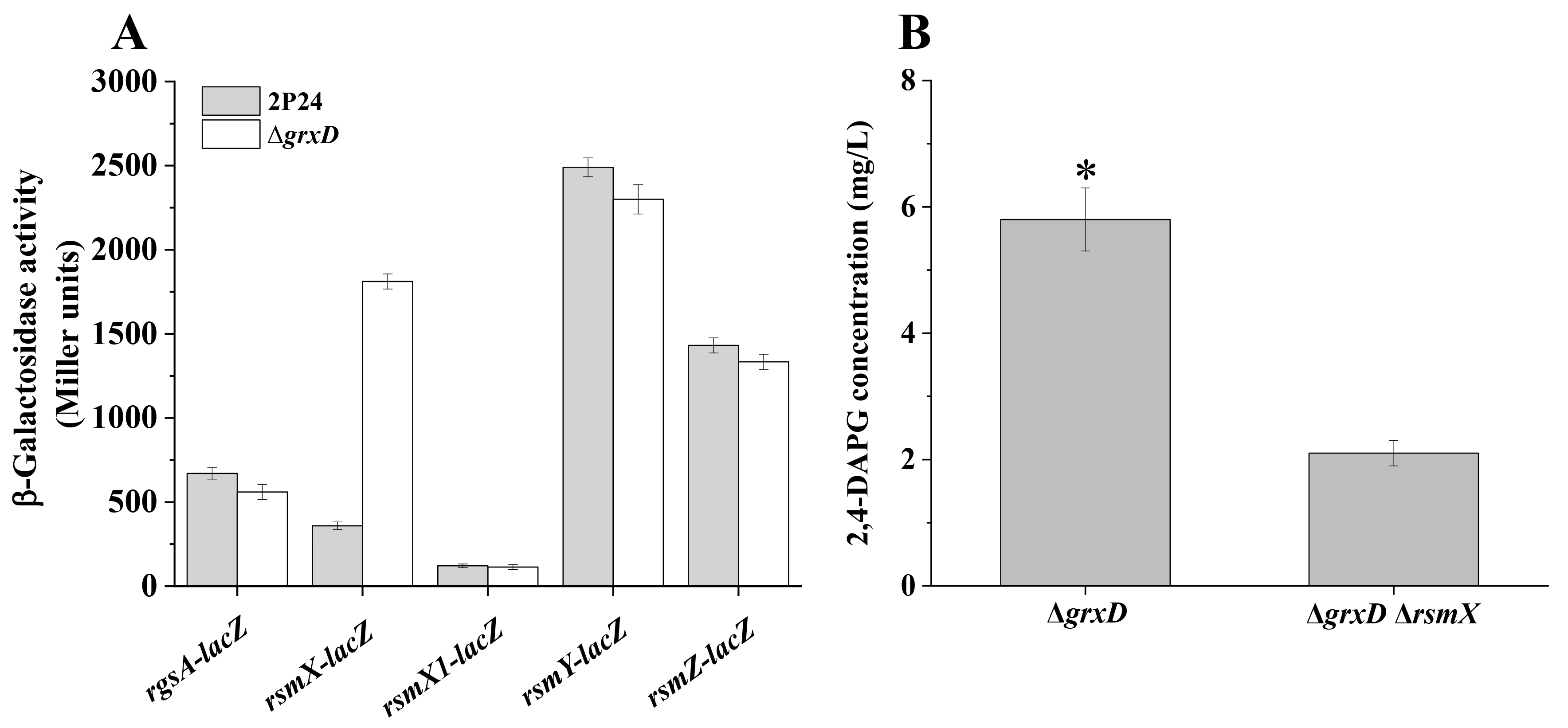
Fig. S3. GrxD influenced the expression of *rgsA*, *rsmX*, *rsmX1*, *rsmY*, and *rsmZ*. (A) The expression of *rgsA*-*lacZ*, *rsmX*-*lacZ*, *rsmX1*-*lacZ*, *rsmY*-*lacZ*, and *rsmZ*-*lacZ* transcriptional fusions was determined in strain 2P24 and the *grxD* mutant at 36 h after inoculation. (B) Quantification of the production of 2,4-DAPG from the *grxD* mutant and the *grxD rsmX* double mutant. All experiments were performed in triplicate, and the mean values ± standard deviations are indicated, **P* < 0.05.


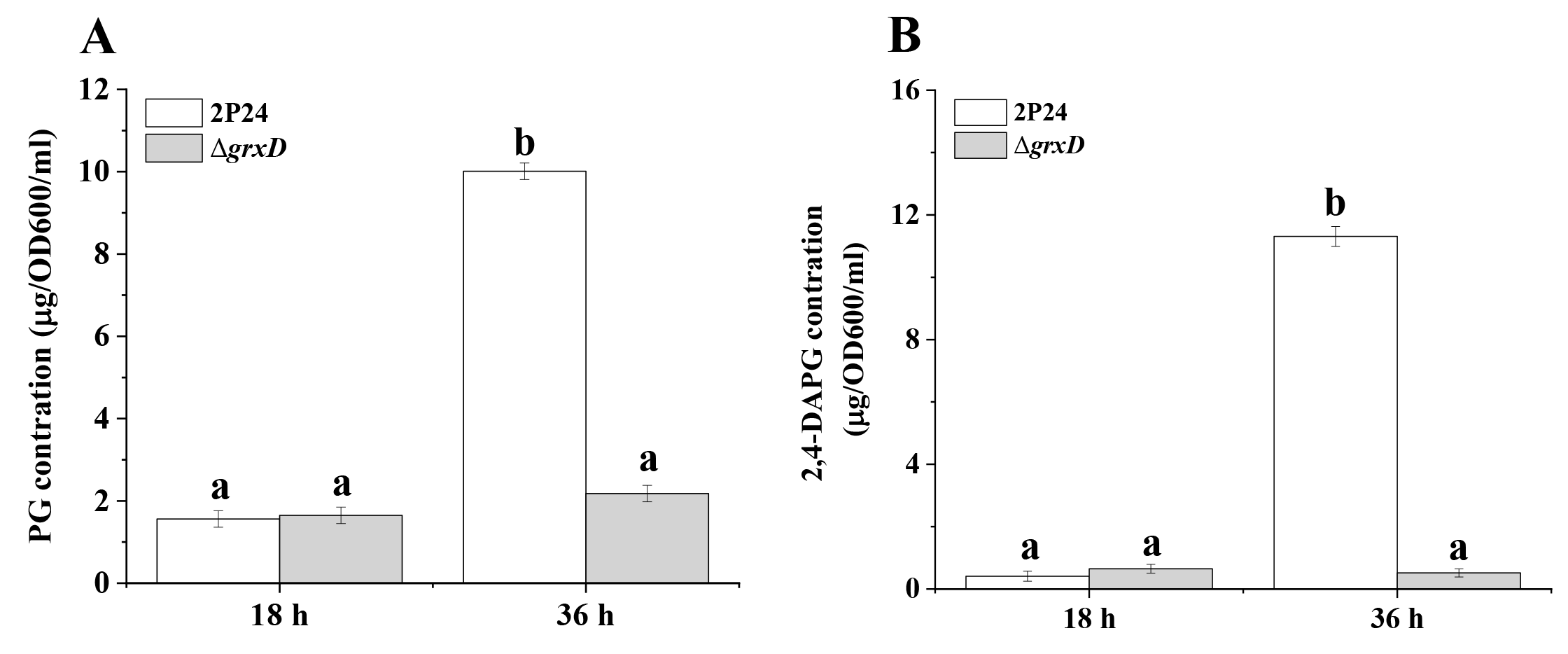
Fig. S4. GrxD influenced the production of PG (A) and 2,4-DAPG (B) of strain 2P24 at 18h and 36 h after inoculation. All experiments were performed in triplicate, and letter represents significant differences between samples where *P* < 0.05.


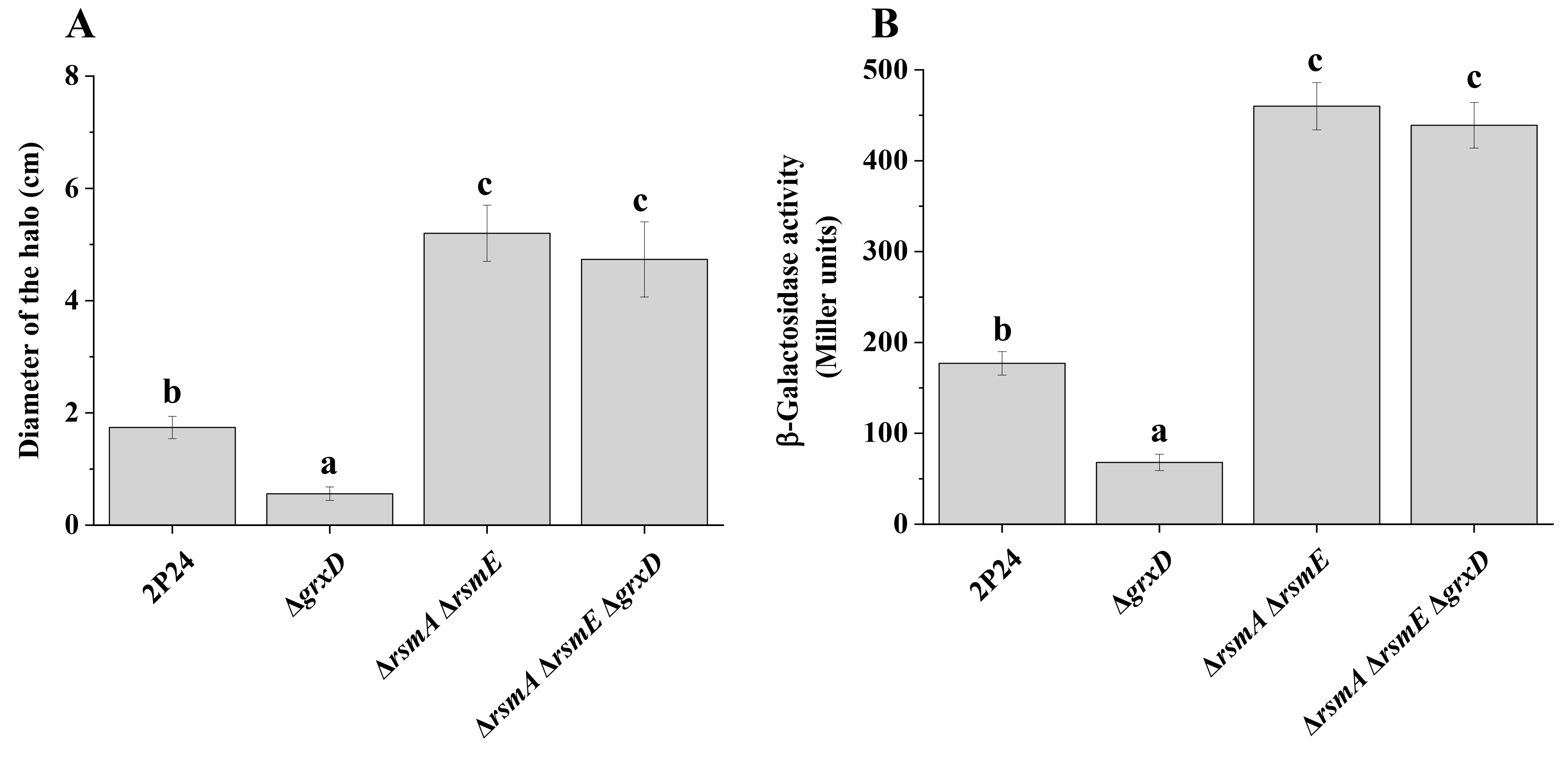


Fig. S5. GrxD influenced the production of protease in strain 2P24. (A) Protease production of strain 2P24 and its derivatives was measured by the diameter of the orange circle on the CAS plate. (B) The expression of *aprA′*-*′lacZ* translational fusion was determined in strain 2P24 and the *grxD* mutant. All experiments were performed in triplicate, and letter represents significant differences between samples where *P* < 0.05.


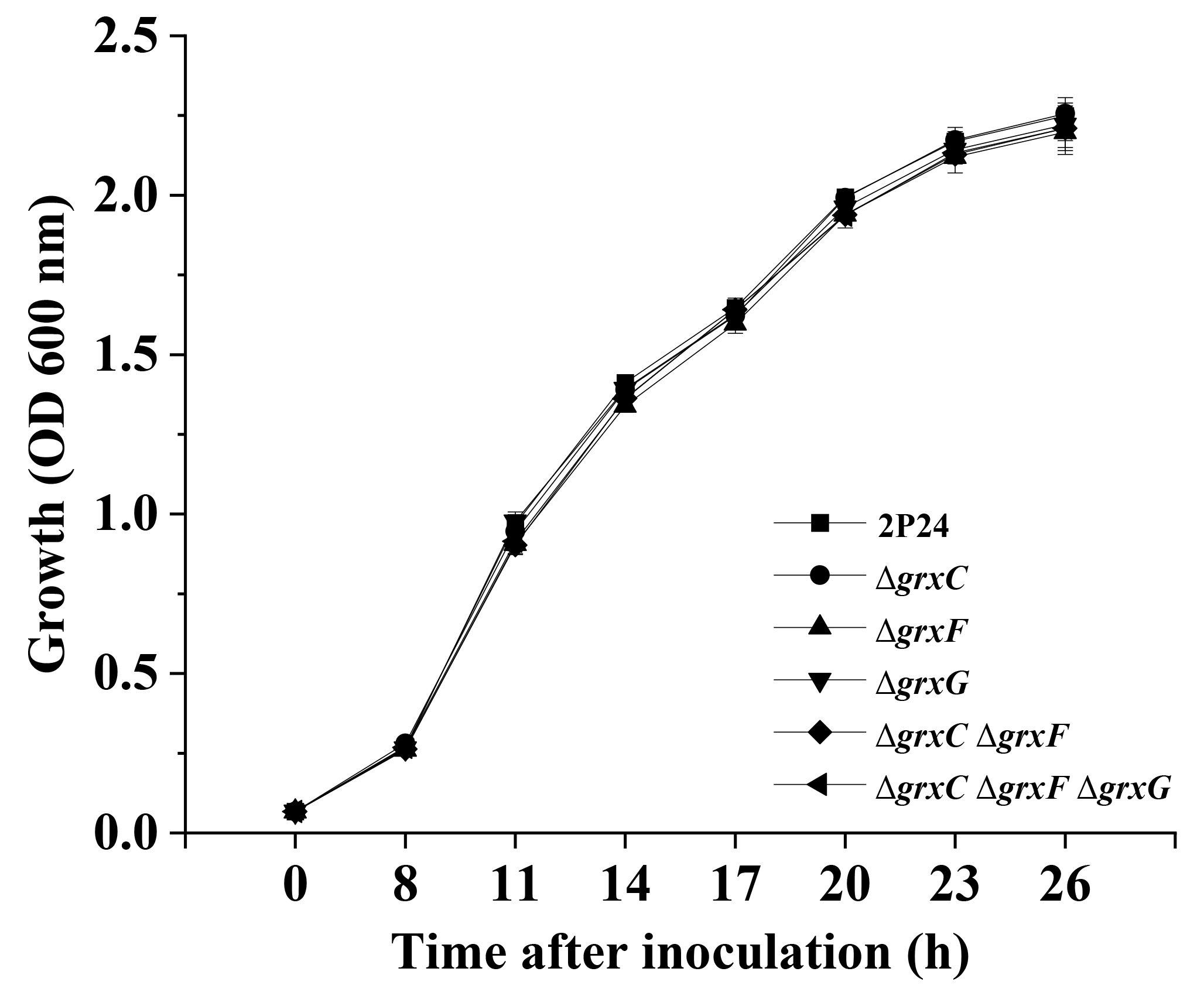


Fig. S6. The effect of *grx* genes on the growth of *P. fluorescens*. Strain 2P24 and its derivatives were cultured in LB medium and the absorbance at 600 nm were measured at different points of the growth curve. All experiments were performed in triplicate, and different letters indicate a significant difference at the 0.05 level.
